# Myopericytoma on the nasal turbinate

**DOI:** 10.1093/jscr/rjaf1059

**Published:** 2026-01-15

**Authors:** Jacob S Gervais, Veena V Vats, Ishani R Vats, Guangming Guo, Naveen V Kumar

**Affiliations:** Trinity ENT and Facial Aesthetics, 1760 E Pecos Rd, Ste 401, Gilbert, AZ 85295, United States; Trinity ENT and Facial Aesthetics, 1760 E Pecos Rd, Ste 401, Gilbert, AZ 85295, United States; Trinity ENT and Facial Aesthetics, 1760 E Pecos Rd, Ste 401, Gilbert, AZ 85295, United States; ClinPath Diagnostics, 4313 E Cotton Center Blvd, Ste 120, Phoenix, AZ 85040, United States; Trinity ENT and Facial Aesthetics, 1760 E Pecos Rd, Ste 401, Gilbert, AZ 85295, United States

**Keywords:** myopericytoma, nasal turbinate, haemangiopericytoma, perivascular tumour

## Abstract

Myopericytoma is a mesenchymal tumour that shares characteristics with other soft tissue tumours including glomus tumours and myofibromas. These pericytic tumours show characteristic perivascular growth patterns (Sbaraglia M, Bellan E, Dei Tos AP. The 2020 WHO classification of soft tissue tumours: news and perspectives. *Pathologica* 2021;**113**:70–84. https://doi.org/10.32074/1591-951X-213). Myopericytoma differentiates itself as it tends to have a spindle shape histologically, rather than an epithelioid shape as with the glomus variety. Myopericytoma rarely demonstrate malignant behaviour. A 67-year-old male presented to our clinic with complaints of increased frequency of right sided epistaxis for several months. The epistaxis episodes were controlled with oxymetazoline spray and manual pressure to the nasal tip. Office nasal endoscopy revealed a mass emanating from the anterior end of the right inferior turbinate. Complete excision was performed endoscopically. Histopathological analysis revealed myopericytoma, a rare tumour typically arising from the epithelium or submucosa. This case underscores the need for otolaryngologists to consider perivascular tumours in the differential diagnosis of intranasal masses as complete excision is recommended to avoid recurrence.

## Introduction

Myopericytoma (MPC) is a rare soft tissue tumour accounting for 1% of all vascular tumours, 25% of which are in the head and neck [1, 2]. Occurrence in the sinonasal region is rare. ‘Fewer than 200 cases of MPC have been reported in the literature to date’ [13] making this case an important addition to our understanding of sinonasal vascular tumours. MPC are most common in middle-aged males. They are often discovered in distal lower extremities or the digits, arising in the epithelium or submucosa [3]. MPC is a type of perivascular tumour, a category which also includes glomus tumour, myofibroma, and angioleiomyoma. Since 2013, the older classification of haemangiopericytoma has been removed by the WHO allowing for a more precise classification of perivascular tumours. The 2020 WHO system organized soft tissue tumours into distinct categories such as adipocytic, vascular, and notably pericytic (perivascular) tumours, which now includes MPC [1]. Tumours are now further subclassified based on behavior: benign, intermediate (locally aggressive or rarely metastasizing), and malignant [1]. Complete resection is the primary treatment with radiotherapy reserved for the rare aggressive recurrent invasive malignancies.

## Case presentation

**Figure 1:**
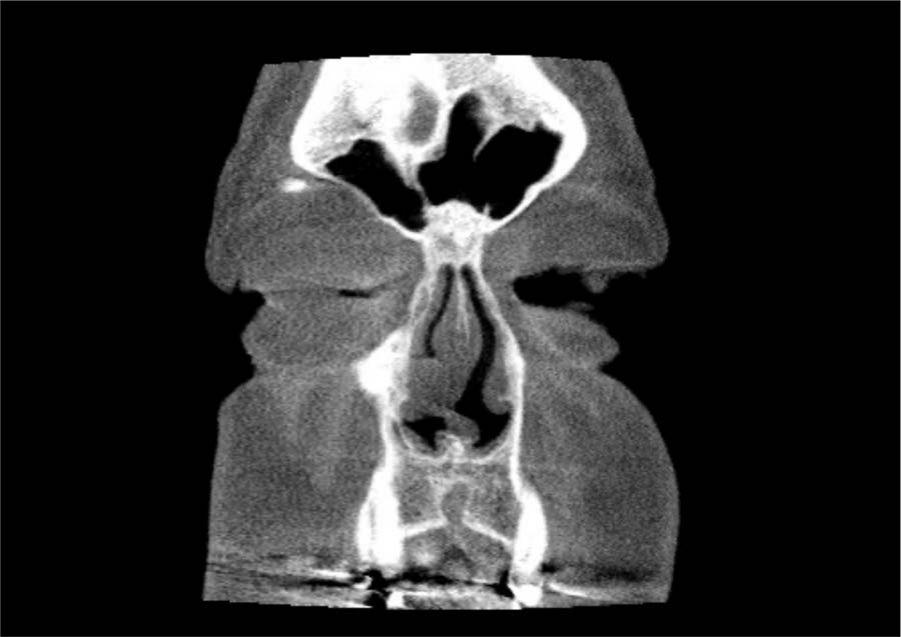
Coronal non-contrast CT scan images of soft tissue lesion on right inferior turbinate.

**Figure 2:**
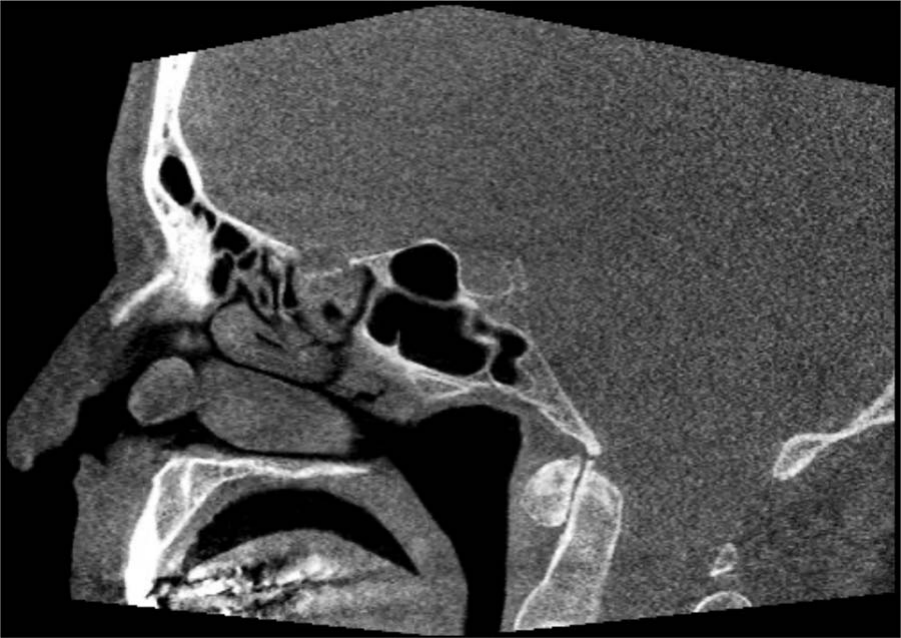
Sagittal non-contrast CT scan images of soft tissue lesion on right inferior turbinate.

**Figure 3:**
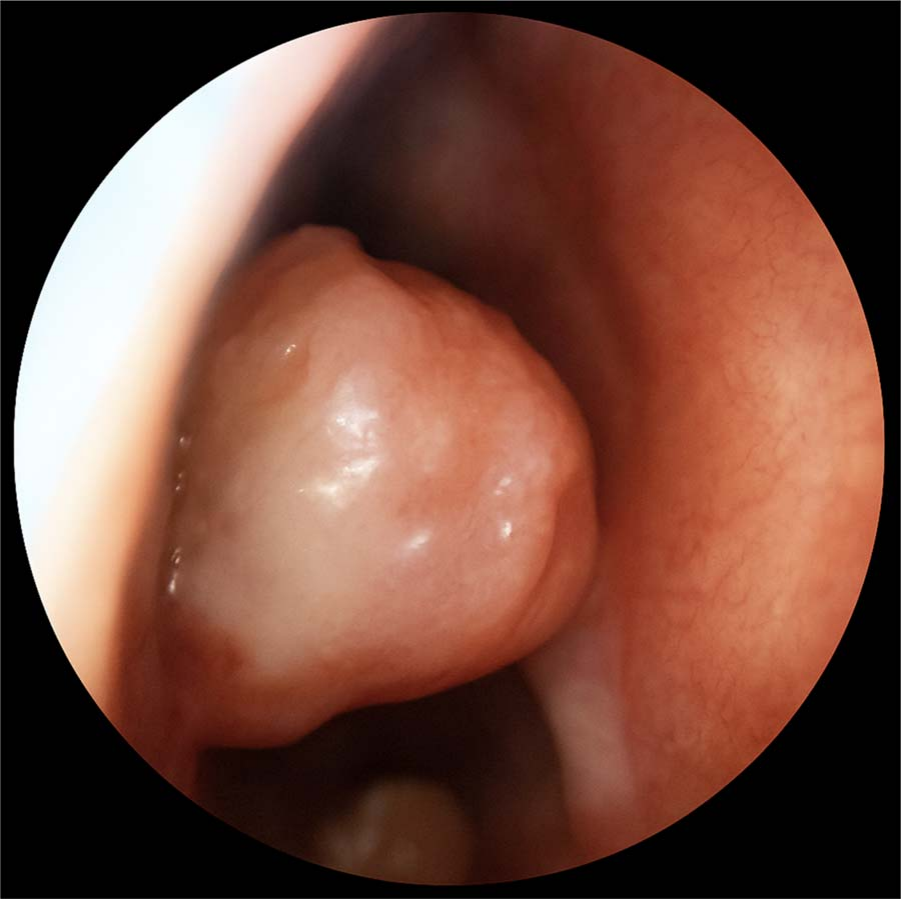
Nasal endoscopy depicting lesion in caudal right nasal cavity.

A 67-year-old male presented to our outpatient office with complaints of increasing bouts of right sided nasal bleeding for the preceding twelve months. He had been able to control the epistaxis with pressure and oxymetazoline nasal spray; however, a recent Emergency Room visit for persistent nasal bleeding prompted a referral to our office. The patient denied nasal trauma or other sinonasal problems in the past or present. He was not taking any anticoagulant or antiplatelet therapies. Nasal endoscopy revealed a soft, pale, non tender mass on the caudal end of the right inferior turbinate. Coagulated blood was noted over the anterior portion of this mass, without any vascular prominence or bleeding appreciated over the septal wall. This indicated the nasal mass was the site of recurrent epistaxis. Non-contrast computed tomography (CT) of the sinuses (Figs 1 and 2), showed a soft tissue prominence at the same area seen on endoscopy (Fig. 3).

Differential diagnosis included sinonasal inflammatory polypoid disease, nasal papilloma, sinonasal haemangioma, malignancy, amongst others with a glomus tumour lower on the differential given the rate of occurrence in the nasal cavity. The mass was excised using scissors at its broad base. An oscillating shaver blade was used on the anterior end of the turbinate and an absorbable sponge was placed for hemostasis.

**Figure 4:**
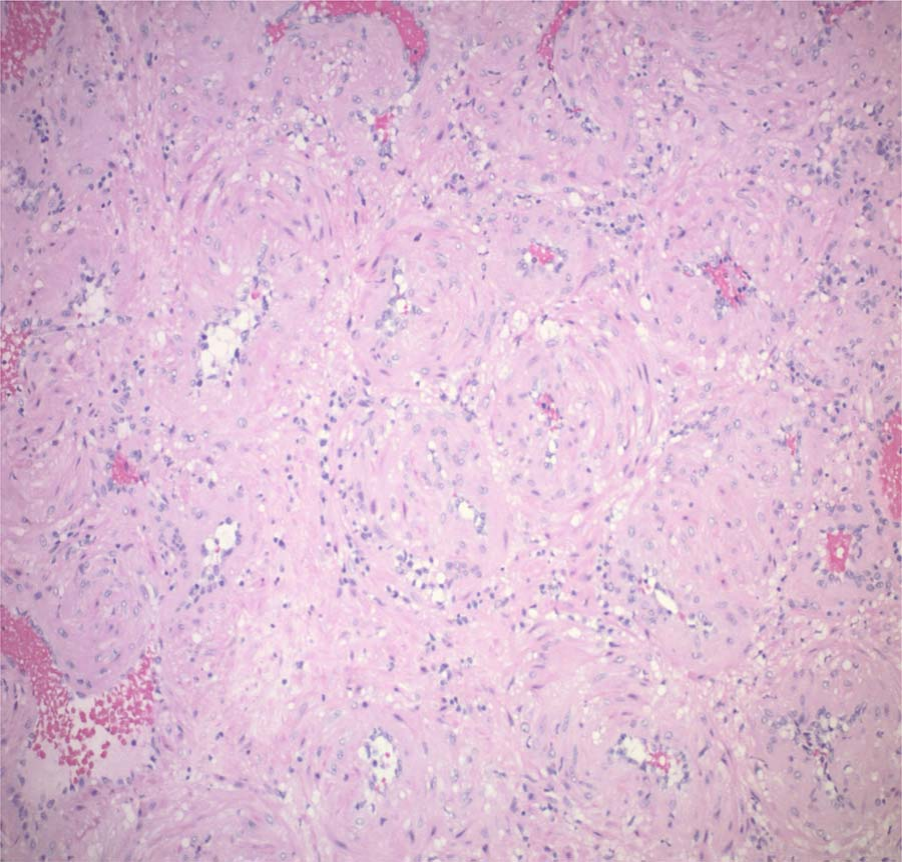
The tumour is composed of bland myoid spindle cells growing in a concentric pattern around numerous small vessels.

**Figure 5:**
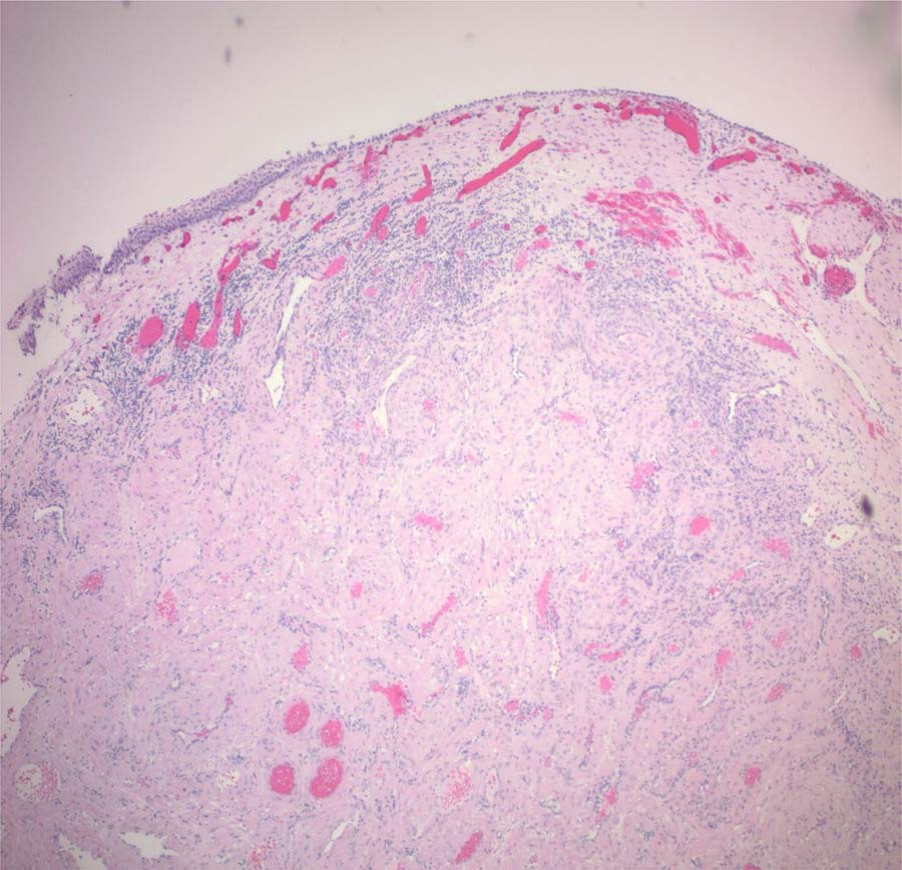
Tumour is unencapsulated, involving mucosa.

Histopathological analysis showed a 1.2 cm tan pink mass composed of bland myoid spindle cells growing in a concentric pattern around numerous small vessels (Fig. 4). There was no mitotic activity, cytologic atypia or necrosis present (Fig. 5). Immunohistochemical stain showed that the tumour cells were positive for smooth muscle actin (SMA) while negative for Desmin. CD34 highlighted the vascular structures within the tumour but was negative in the myoid cells. An Epstein-Barr encoding region in situ hybridization (EBER ISH) study was performed and was negative. The patient was seen for 1 and 3 week follow up with appropriate healing of the nasal mucosa and reports of no recurrent epistaxis episodes following procedural intervention.

## Discussion

MPC is a rare, often benign mesenchymal tumour typically arising in the extremities as a slow growing painless mass [4]. Occurrence in the head and neck is uncommon accounting for <25% of MPC and are even rarer in the sinonasal region [5]. MPC can occur at any age although they seem to occur more frequently in men and in the sixth to seventh decade of life [6, 7]. The tumours are usually well circumscribed and unencapsulated necessitating complete resection to avoid recurrence [8, 9]. Additionally, the clinician should consider other risk factors that may contribute to recurrence of the tumour including positive surgical margins or immunosuppression. MPC are typically slow growing with low mitotic activity and solitary, although local bony invasion and metastasis have been reported [6]. Malignant potential seems higher in tumours > 5 cm, increased mitotic activity, and necrosis [6]. Histologically, MPC is characterized by bland oval to spindle shaped cells with eosinophilic cytoplasm arranged in concentric perivascular pattern [5]. MPC arise from pericyte cells surrounding blood vessels. The pericyte cell lies along capillaries and venules with extensions around the vessels, and is thought of as a pluripotent stem cell, capable of differentiating along smooth muscle, osseous, pericyte, glomus, adipose, and fibroblast cell lines [10]. Thus, these perivascular myoid cells share characteristics with both smooth muscle and glomus cells [3]. Further, MPC can share similar characteristics with glomus tumour, myofibroma, and angioleiomyoma [1]. Indeed, MPC and myofibromas can easily be mistaken for sarcomas [3, 11]. However, with the removal of haemangiopericytoma from nomenclature, perivascular tumours can be more precisely identified. Haemangiopericytoma had been used to describe a variety of solid fibrous tumours including MPC, myofibroma, synovial sarcoma, endometrial stroma sarcoma, peripheral nerve sheath tumour, and mesenchymal chondrosarcoma [1].

Histoplathological analysis determines the diagnosis. MPC features include well circumscribed and unencapsulated lobular mass typically <2 cm in size. Cells are spindle or ovoid shaped in MPC with eosinophilic cytoplasm and bland nuclei. Mitoses are rare. Blood vessels are numerous and the myoid cells are multilayered in a concentric pattern around the thin-walled vessels. Staining is positive for SMA and h-caldesmon. Desmin, CD31, CD34, S100, keratin, and STAT 6 should be negative, although focal desmin positivity can be seen [12]. In our patient’s specimen, the tumour is clearly seen in the submucosa extending into the mucosa.

## Conclusion

This case illustrates the importance of including vascular tumours like MPC in the differential diagnosis of intranasal lesions, especially when patients present with unexplained epistaxis emanating from visible nasal masses. Histologic diagnosis, which is supported by immunohistochemistry, is critical to distinguish a MPC from morphologic mimics such as glomus tumours, myofibromas, and haemangiopericytoma. Complete surgical excision remains the cornerstone of treatment, followed by long-term surveillance given the tumour’s potential for recurrence or local invasion. Early recognition by otolaryngologists can prevent misdiagnosis and ensure timely, effective intervention.
